# Maintaining momentum: the history and future of lung cancer disparities in the United States

**DOI:** 10.3389/fonc.2026.1799293

**Published:** 2026-05-08

**Authors:** Krista Casazza, Emma N. Wickland, Jennifer Zhu, Robert A. Winn

**Affiliations:** Massey Comprehensive Cancer Center, Virginia Commonwealth University, Richmond, VA, United States

**Keywords:** health disparities, lung cancer, lung cancer screening, mortality trends, precision oncology

## Abstract

Lung cancer, once considered a clinical rarity, is now the leading cause of cancer-related mortality among both men and women in the United States (US) and remains the deadliest cancer worldwide. Early investigations focused on a broad range of environmental and occupational exposures as putative factors. However, the emergence of robust epidemiologic evidence in the mid-twentieth century established tobacco use, particularly cigarette smoking, as the dominant driver of lung cancer incidence. These discoveries fundamentally shaped US tobacco control policies, cancer prevention strategies, and subsequent scientific advances in thoracic oncology. Over the past three decades, innovations in screening, staging, molecular characterization, and treatment have transformed the landscape of lung cancer care and created unprecedented opportunities to improve outcomes for populations at greatest risk. Yet, the benefits in these advancements have not been equally realized. Persistent disparities in access to screening, prevention, timely diagnosis, and evidence-based care continue to disproportionately burden at-risk groups, including individuals with lower socioeconomic status and those in rural areas. Addressing these inequities requires multifaceted, cross-sector strategies that extend beyond the clinic to the social and structural determinants of health. This review aims to provide a brief history of lung cancer research, reflect on advancements in lung cancer treatment from 1990 - 2025, and highlight efforts toward continued progress in truly equitable lung cancer screening, treatment, care, and policy.

## Introduction

Lung cancer remains the leading cause of cancer-related morbidity and mortality worldwide. In 2022, an estimated 20.0 million new cases and 9.7 million deaths were attributed to the disease globally (1). In the United States (US), lung and bronchus cancer is the second most commonly diagnosed malignancy in men and women but continues to account for more cancer deaths than any other cancer type (2, 3). Although substantial progress has been made in prevention, early detection, and treatment over the past three decades, lung cancer continues to impose a significant public health burden.

Cigarette smoking remains the predominant driver of lung cancer incidence, responsible for the majority of lung cancer deaths in the contemporary US population (4). However, lung cancer risk reflects the intersection of behavioral, environmental, and biological factors. A growing proportion of lung cancer cases occur among individuals who have never smoked, particularly among women and populations in high–human development index settings, highlighting contributions from environmental exposures, occupational hazards, and genetic susceptibility (1, 4).

Over the past several decades, lung cancer incidence and mortality have declined in many high-income countries, reflecting reductions in smoking prevalence and advances in early detection and treatment. Despite these gains, substantial disparities persist across racial and ethnic groups, socioeconomic strata, and geographic regions. These inequities manifest across the continuum of care from exposure to risk factors and eligibility for screening to access to diagnostic evaluation, biomarker testing, and state-of-the-art therapies. This review examines lung cancer through a disparities-focused lens. Specifically, we trace the historical development of lung cancer epidemiology and control, summarize major advances in prevention, screening, and treatment, and highlight persistent inequities in incidence, diagnosis, and outcomes. By integrating epidemiologic, clinical, and policy perspectives, we aim to identify opportunities for research, public health action, and community engagement that can sustain mortality reductions while promoting more equitable outcomes in thoracic oncology.

## Historical overview

Prior to the turn of the twentieth century, lung cancer was considered a rare disease. Early medical reports documented relatively few cases annually in the US, and the disease was initially attributed primarily to occupational and environmental exposures such as arsenic, coal tar, radon, and asbestos (5–8). During this period, tobacco was already widely cultivated and consumed in various forms, but cigarette use remained relatively limited until the late nineteenth century. The industrialization of cigarette production in the 1880s, enabled by the introduction of the automatic cigarette-rolling machine, dramatically expanded cigarette availability and consumption (9, 10). Over the following decades, cigarette smoking became increasingly widespread, fueled by mass marketing, cultural shifts during wartime, and aggressive advertising campaigns that often portrayed smoking as socially desirable or even beneficial to health (5, 9, 11, 12). The first formal suggestion of a link between cigarette smoking and lung cancer is often attributed to Isaac Adler, who in 1912 noted a rising incidence of lung tumors coinciding with increasing cigarette use (8, 13). More definitive evidence emerged in the mid-twentieth century through landmark epidemiologic studies. In 1950, Richard Doll and Austin Bradford Hill published a case-control study demonstrating a strong association between cigarette smoking and lung cancer risk, while cohort studies led by E. Cuyler Hammond and Daniel Horn in the early 1950s further confirmed the relationship (14, 15). These findings marked a turning point in the scientific understanding of lung cancer etiology. Public health recognition of the dangers of smoking culminated with the landmark 1964 US Surgeon General’s Report which concluded that cigarette smoking was causally linked to lung and other major chronic diseases (7, 16, 17). The report catalyzed a series of policy interventions, including the Federal Cigarette Labeling and Advertising Act of 1965 and the eventual ban on cigarette advertising on television and radio in 1970 (9). These measures marked the beginning of modern tobacco control efforts and established the foundation for subsequent public health strategies aimed at reducing smoking prevalence and preventing lung cancer.

## Decades of progress in lung cancer control

Over the past three decades, the US has experienced unprecedented improvements in lung cancer outcomes (10). These gains reflect the cumulative effects of tobacco control policies, advances in early detection, rapid progress in molecular oncology, and improvements in multidisciplinary cancer care. Together, these developments have produced measurable reductions in lung cancer mortality that once seemed unattainable. Yet the pace and distribution of these improvements remain uneven across populations, underscoring the importance of evaluating progress through an equity-focused lens.

In the early 1990s, lung cancer mortality in the US reached its historical peak (18). This peak reflected decades of high smoking prevalence, limited early detection capabilities, and the absence of effective systemic therapies for advanced disease (19). Since that time, lung cancer mortality has declined by more than 48% among men and approximately 33% among women, with the most rapid reductions occurring in the past decade (20, 21). Notably, the decline in lung cancer mortality has outpaced that of most other major cancers, reflecting the combined influence of declining tobacco use and transformative therapeutic innovations introduced since the early 2010s (1, 22). Between 1991 and 2021, reductions in lung cancer mortality accounted for nearly half of all cancer deaths averted in the US, highlighting the outsized contribution of lung cancer control efforts to national cancer mortality trends (21).

Comprehensive tobacco control policies remain the single most important contributor to these mortality reductions (23). Cigarette consumption in the US has fallen by more than 60% since the 1980s as a result of increased taxation, smoke-free legislation, mass media campaigns, warning labels, and expanded access to smoking cessation services (24). These interventions substantially reduced smoking prevalence across the population and ultimately lowered lung cancer incidence. However, tobacco control efforts also revealed persistent structural inequities in exposure and prevention. For decades, tobacco industry marketing strategies disproportionately targeted Black communities and low-income populations, particularly through promotion of menthol cigarettes and other products (25–27). Rural populations have also experienced persistently higher smoking prevalence and heavier tobacco use compared with urban populations, reflecting differences in policy environments, economic conditions, and healthcare access (28, 29). While overall smoking rates have declined, these disparities in tobacco exposure continue to influence patterns of lung cancer risk and disease burden.

Parallel advances in early detection have also contributed to improved outcomes. Early attempts at lung cancer screening in the mid-twentieth century focused primarily on chest radiography and sputum cytology. Randomized controlled trials (RCTs) conducted in the 1960s and 1970s, including (but not limited to) studies carried out by the Northwest London Mass Radiography Service and several National Cancer Institute (NCI)–funded trials at Memorial Sloan Kettering, Johns Hopkins, and the Mayo Clinic, evaluated combinations of chest radiographs and sputum cytology in high-risk populations. Although these studies demonstrated increased detection of early-stage tumors, they failed to show significant reductions in lung cancer mortality (30–33). Methodological limitations, including relatively small sample sizes and the use of chest radiography within control groups, complicated interpretation of these findings and slowed progress in screening research. In 1993, the NCI initiated the Prostate, Lung, Colorectal, and Ovarian (PLCO) Cancer Screening Trial to further evaluate the role of chest radiography in cancer detection. Among nearly 155,000 participants, annual chest radiograph screening over four years did not significantly reduce lung cancer mortality compared with usual care (10, 30, 34). These results reinforced the limitations of conventional radiographic screening approaches and prompted investigation of more sensitive imaging technologies.

The introduction of low dose computed tomography (LDCT) screening marked a major turning point in lung cancer detection. In the late 1990s and early 2000s, observational studies demonstrated that LDCT could identify substantially more early-stage lung cancers than chest radiography. These findings would inspire NCI’s landmark National Lung Screening Trial (NLST) in the US, the largest RCT involving nearly 54,000 high-risk current or former smokers to date, which showed that LDCT could significantly reduce mortality related to lung cancer (35, 36). Other major RCTs, such as the Dutch-Belgian lung-cancer screening trial (Nederlands–Leuvens Longkanker Screenings Onderzoek, also known as NELSON) in Europe also demonstrated these results and affirmed how LDCT screening could substantially improve early lung cancer detection compared with conventional approaches (37). These findings contributed to the evidence base for modern lung cancer screening and informed recommendations by the US Preventive Services Task Force (USPSTF), which first endorsed annual LDCT screening for high-risk populations in 2013 and later expanded eligibility criteria in 2021 to include younger individuals and those with lower cumulative smoking exposure (36, 38). These guideline expansions were particularly important for improving screening eligibility among Black smokers and individuals of lower socioeconomic status who tend to develop lung cancer at younger ages and with fewer pack-years of smoking (39, 40).

Although the adoption of LDCT screening represents a major advance in early detection, national uptake remains low (41). Fewer than 10% of eligible individuals in the US currently undergo screening annually (42). Nevertheless, screening programs implemented within integrated healthcare systems and safety-net settings have begun shifting stage distribution toward earlier diagnosis, with a growing proportion of lung cancers detected at stages amenable to curative treatment (43). While advances in screening were emerging, the field of lung cancer biology underwent a profound transformation with the advent of molecular profiling. Histological patterns of lung cancer have shifted substantially over recent decades. While squamous cell carcinoma and small cell carcinoma have declined alongside reductions in smoking prevalence, adenocarcinoma has become the dominant histologic subtype worldwide, particularly among women and individuals who have never smoked. These changes parallel modifications in cigarette design, including filtered and low-tar formulations that facilitate deeper inhalation and alter the pattern of carcinogen deposition in peripheral lung tissue.

Molecular characterization of lung tumors further revealed that a growing subset of lung cancers, particularly among people who have never smoked (44), women, and certain racial and ethnic groups, are driven by actionable oncogenic alterations (45). These include mutations in the epidermal growth factor receptor (EGFR) gene, rearrangements involving the anaplastic lymphoma kinase (ALK) gene, and fusions involving the ROS1 receptor tyrosine kinase (1, 4, 46). The identification of these driver alterations fundamentally reshaped the understanding of lung cancer pathogenesis and led to the development of highly effective targeted therapies. Beginning in the early 2010s, tyrosine kinase inhibitors targeting EGFR, ALK, and ROS1 dramatically improved outcomes for biomarker-defined patient populations. For individuals with EGFR-mutated non–small cell lung cancer, first-line EGFR inhibitors have extended median survival beyond three years in advanced disease, transforming what was once a uniformly fatal malignancy into a chronic, molecularly modulated condition for many patients (47, 48). More recently, targeted agents directed at additional genomic alterations (e.g., KRAS G12C mutations) have further expanded therapeutic options.

Immunotherapy has also played a transformative role in lung cancer treatment. Between 2015 and 2020, immune checkpoint inhibitors targeting the PD-1/PD-L1 pathway (e.g., pembrolizumab, nivolumab, atezolizumab) demonstrated significant survival benefits across multiple treatment settings. These therapies produced unprecedented improvements in long-term survival for patients with advanced non–small cell lung cancer and are now widely integrated into first line and adjuvant treatment strategies (49). Importantly, real-world evidence suggests that when access to immunotherapy is equitable, survival improvements occur across racial and ethnic groups, highlighting the importance of addressing structural barriers to care rather than attributing outcome differences to biological variation alone (50).

Technological advances in local treatment modalities have further expanded curative options for patients with early-stage disease or significant medical comorbidities. Minimally invasive thoracic surgical approaches, (e.g., video-assisted thoracoscopic surgery, robotic-assisted techniques) have reduced perioperative morbidity and expanded the population of patients eligible for surgical resection. In parallel, stereotactic ablative radiotherapy has emerged as an effective non-surgical alternative for medically inoperable early-stage lung cancer.

Improvements in cancer care delivery have also contributed to improved outcomes. Multidisciplinary care models, rapid diagnostic pathways, and patient navigation programs have been shown to reduce delays in diagnosis and treatment initiation, particularly among patients from historically marginalized communities (51, 52). Molecular tumor boards and expanded access to comprehensive biomarker testing have further facilitated the implementation of precision oncology in routine clinical practice (53).

Despite these remarkable advances, significant disparities in lung cancer outcomes persist. Although lung cancer mortality has declined across racial and ethnic groups in the US, the magnitude and pace of improvement remain uneven (54, 55). In the 1990s, Black men experienced lung cancer mortality rates nearly 40% higher than those of White men; although this gap has narrowed substantially over time, Black men still experience higher mortality rates overall (56–58). American Indian and Alaska Native populations, particularly those living in the Northern Plains, continue to experience some of the highest lung cancer mortality rates nationally, reflecting persistent structural, geographic, and environmental inequities (59–61). This persistent disparity reflects a complex interplay of factors across the cancer care continuum, including differences in cumulative tobacco exposure and menthol cigarette use, lower likelihood of meeting traditional screening eligibility criteria, reduced uptake of LDCT screening, and delays in diagnosis leading to more advanced-stage presentation (62). In addition, Black populations are less likely to receive guidance-concordant staging, curative-intent surgery, comprehensive biomarker testing and timely access to novel systemic therapies (63, 64). Hispanic and Asian American populations generally have lower overall incidence and mortality rates, yet later-stage diagnosis remains common due to linguistic, cultural, and healthcare access barriers. In addition, lung cancer among those who have never smoked occurs disproportionately among Asian women, in part because of the higher prevalence of actionable genomic alterations such as EGFR and ALK in this population (65, 66). Taken together, the past several decades represent significant periods of progress in the history of lung cancer control. The dramatic nationwide decline in mortality reflects the combined effects of effective tobacco control policies, advances in early detection, and the rapid evolution of targeted and immunotherapeutic treatments. However, the persistence of disparities in smoking exposure, screening uptake, diagnostic timeliness, biomarker testing, and access to innovative therapies underscores a central lesson of modern lung cancer control: scientific breakthroughs reduce mortality, but equitable implementation determines who benefits from these advances.

## The power of research and community

The extensive progress that has been made in improving overall lung cancer outcomes and reducing disparities in recent decades has not occurred in isolation. These advances are the result of sustained efforts by researchers, clinicians, public health practitioners, community leaders, and advocates that have worked collaboratively to transform prevention, detection, and treatment. However, even with tremendous overall progress, disparities between socioeconomic status, location, race and ethnicity, education attainment, gender, age, occupation, and other demographics have remained (67–71). Just as much as one’s DNA affects a person’s risk of cancer, ZNA, or one’s zip code and neighborhood of association, can play a large part (72). The residential segregation that leads to Black and lower income populations living in areas with greater pollution greatly affects their lung cancer risk (73, 74). Recent evidence further demonstrates that structural systems beyond environmental exposure, such as county-level incarceration are independently associated with elevated lung cancer mortality. Sarfraz et al. (2025) reported high incarcerations counties experience 7-10% higher deaths, disproportionately affecting Black residents (75). Since incarceration in the US is associated with increased tobacco use (76), elevated environmental pollutants (incarcerated individuals are more likely to be held near mines or industrial sites), aging infrastructure that is exacerbated by extreme weather, and poor ventilation (77), it is possible that these factors contribute to the increased lung cancer mortality in incarcerated individuals. As society works to reduce emissions and improve disposal methodologies, improving sustainable and equitable access to lung cancer screening, treatment, and survivorship services (e.g., insurance, transportation, language, food, housing, screenings and treatments in community settings, and childcare, among other supportive approaches to cancer care) can help to bridge these disparities (78).

With a rise in lung cancer incidence for people who have never smoked across demographics (79), it is important to continue with basic and clinical research to determine the potential cause of these increases, and to develop better treatments for the different types of lung cancer. Due to the close tie between lung cancer and modifiable risk and environmental factors, it is imperative that we work across silos with social scientists, science communicators, environmental engineers, climate scientists, and policymakers to tackle this issue together.

Just as tobacco control strategies greatly impacted the number of lung cancer deaths in the late twentieth century (80), strategies that help reduce pollutants like particulate matter and radon have the capacity to further improve lung cancer outcomes (81, 82). In addition, policies that address accelerating climate change also have the potential to improve lung cancer outcomes. Climate change increases extreme weather events (83) like wildfires that can release carcinogens into the environment. Extreme weather events can also disrupt routine healthcare visits like lung cancer screenings (84), damage healthcare infrastructure, and impact access to essential care materials (85). As lung cancer screening is an important pillar for improving patient outcomes, it is imperative that we invest in prevention along every avenue (86, 87). Climate change can also cause longer and drier wildfire seasons (88, 89), increasing the amount of organic and man-made particulates released into the air (90), which has been shown to affect non-small cell lung cancer (91). However, tobacco use is still the leading risk factor contributing to lung cancer (92), and as use of tobacco products in youths have increased recently (93), it is important to continue pursuing tobacco prevention and cessation strategies to further reduce tobacco related lung cancer mortality.

Emerging evidence also suggests that current lung cancer screening eligibility criteria may have differential sensitivity across self-reported race and sex, potentially leading to under-identification of high-risk individuals in certain populations (94–96). This has prompted growing interest in alternative approaches, such as risk-prediction models that incorporate demographic, clinical, and exposure-related factors, as well as guideline modifications that may better capture individuals who develop lung cancer at younger ages or with lower cumulative smoking exposure (97, 98). Addressing persistent disparities will also require more diverse and representative research datasets and clinical trials. Future studies would benefit by the prioritization of inclusive enrollment and the development of data resources that integrate detailed behavioral and environmental exposures, social drivers of health, tumor genomic profiling, and real-world clinical outcomes (99, 100). Such comprehensive datasets are essential for understanding the multi-level drivers of disparities and for designing interventions that promote equitable lung cancer prevention, screening, and treatment.

With federal funding cuts for research that have the capacity to affect lung cancer risk and outcomes (101), there is a need to look toward other funding sources like private foundations, philanthropic organizations, corporate funding, and venture capital. Taking advantage of public-private partnerships (PPPs) to bridge gaps in funding, take advantage of combined resources, and build collective intelligence not only helps to mitigate funding loss, but combine organizations that might not previously have worked together for a common purpose. Examples such as LungMAP and Pragmatica-Lung help to break down siloes and show that collaborating with shared resources can create a force that is greater than the sum of each individual part (102).

There are also lessons learned from global scientific funding strategies. A recent analysis on international research funding systems from the United Kingdom found that certain strategies can further stretch investment: Funding long-term, high-risk, high-reward research, supporting university-industry engagement, funding both basic research as well as applied research, and streamlining funding structure to maximize the money that is going towards scientific pursuits (103).

Globally, lung cancer and other lung health have been identified as an important priority. The 2025 World Health Organization decision, *Promoting and prioritizing an integrated lung health approach* (104), noted that tobacco and radon exposure still need to be continuously addressed, and highlighted the importance of destigmatization and access to screening and treatments. With this common goal, international collaboration is also a way to utilize our collective resources to improve lung cancer outcomes.

As we move forward in further reducing lung cancer burden and disparities, and improving lung cancer mortality rates, it is imperative to keep up the momentum. Investment in improving access to current screening and treatments can help further reduce disparities and lay the foundation for improved access to new technologies as they are created. Removing resources from the continuum of cancer care and beyond has the potential to undo much of this progress and widen disparities in cancer care outcomes even further. A summary of challenges and opportunities to improve lung cancer care and outcomes for all are listed in **Table 1**.

**Table 1:** Current barriers and framework for future progress in lung cancer equity.

Challenges to Lung Cancer Care and Outcome Equity	Opportunities to Improve Lung Cancer Care and Outcomes for All
Structural - Healthcare System: Barriers to accessing health insurance and healthcare, lack of transparency for healthcare system logistics	• Support community-based screening and care • Reduce redlining • Increase health insurance coverage like Medicare • Provide patient navigator programs
Structural - Financial: Lack of paid time off and childcare services, food and housing insecurity	• Support policy that would encourage screening and treatment access for those without paid time off and who need childcare services • Strengthen social safety nets and food supply chains • Combat food deserts • Increase supply of affordable housing • Strengthen renters protections and expand rental assistance
Cultural: Fear and distrust of the medical establishment, language barriers	• Provide interpretation and translation services • Improve collaboration and engagement with community to encourage trust • Invest in outreach to improve health literacy
Environmental: Pollution and environmental/occupational risks, proximity to screening and treatment locations, neighborhood-level effects on individual cancer risk	• Reduce emissions and improve disposal methodologies • Strengthen workplace protections for those working with carcinogenic materials • Invest in reducing climate change • Reduce occupational respiratory hazards • Provide screenings and treatments in community settings • Reduce redlining • Support criminal justice system reform
Scientific: Reduced federal funding, siloed scientific fields, increasing rates of lung cancer for those who have never smoked	• Look toward other funding opportunities like private foundations, philanthropic organizations, corporate funding, and venture capital • Invest in basic and translational science • Support cross-silo collaborations and silo breaking programs to utilize expertise from all fields • Collaborate via public-private partnerships
Individual Risk Behaviors: Tobacco use	• Expand access to evidence-based tobacco cessation programs • Strengthen population-level tobacco control policies • Integrate cessation services into screening programs and cancer care pathways • Develop tailored programs including racially and ethnically minoritized groups, rural communities and individuals with low socioeconomic status

## Looking toward the future of lung cancer

In the past thirty years, we have seen immense progress in reducing lung cancer mortality through investment in screening, tobacco prevention and cessation, and advancements in treatments. While some gaps in outcomes, such as between Black and White men, have also reduced in this time frame, American Indians/Alaska Natives lung cancer incidence and mortality remain extremely high compared to their White counterparts in the US. Other disparities within different socioeconomic statuses, locations, and occupations still remain, highlighting the need for research and technological advances to be widely accessible to more people. Policies that focus on equitable access to screening and treatments for lung cancer that are already widely accepted as standard of care would go a long way in reducing disparities. Notwithstanding, racial and ethnic populations do not represent homogeneous groups. Within-group heterogeneity can substantially influence smoking behaviors, environmental exposures, healthcare utilization, and ultimately lung cancer risk and outcomes. Future research should incorporate measures of acculturation, language preference, and social context to better characterize these nuanced risk patterns and to inform more culturally responsive prevention and care strategies. As we further develop technologies like precision therapies for lung cancers with different genetic markers, it will be important to consider how policies can help provide equitable access through insurance, clinical trials, and patient support services. If innovation in the technology sector is not met with the same innovation in making sure that these technologies can improve the lives of all, then they will not be used to their fullest potential, leaving investment on the table, both in monetary terms and human lives.

Additional strategies that can support progress in fighting lung cancer include considering how basic, translational, and implementation research, as well as clinical funding can be achieved with limited federal resources. Participating in PPPs to consider the wide array of areas that can be developed to continue reducing lung cancer mortality is a promising avenue. There is capacity to continue reducing lung cancer mortality through collaboration on tobacco prevention and cessation, pollution mitigation, lung cancer screening, treatments, and survivorship. But this can only be done together, by investing in and sustaining innovations in the lab and the clinic, and partnerships in the community.
